# Enhanced Throughput for Electrokinetic Manipulation of Particles and Cells in a Stacked Microfluidic Device

**DOI:** 10.3390/mi7090156

**Published:** 2016-09-01

**Authors:** Lin Zhu, Saurin H. Patel, Mark Johnson, Akshay Kale, Yash Raval, Tzuen-Rong Tzeng, Xiangchun Xuan

**Affiliations:** 1 School of Engineering, Anhui Agricultural University, Hefei 230036, China; zl009@mail.ustc.edu.cn; 2 Department of Mechanical Engineering, Clemson University, Clemson, SC 29634-0921, USA; saurinp@g.clemson.edu (S.H.P.); mhjohns@g.clemson.edu (M.J.); akale@g.clemson.edu (A.K.); 3 Department of Biological Sciences, Clemson University, Clemson, SC 29634-0314, USA; yraval@g.clemson.edu (Y.R.); TZUENRT@clemson.edu (T.-R.T.)

**Keywords:** dielectrophoresis, reservoir, particle separation, particle concentration, parallel operation

## Abstract

Electrokinetic manipulation refers to the control of particle and cell motions using an electric field. It is an efficient technique for microfluidic applications with the ease of operation and integration. It, however, suffers from an intrinsic drawback of low throughput due to the linear dependence of the typically very low fluid permittivity. We demonstrate in this work a significantly enhanced throughput for electrokinetic manipulation of particles and cells by the use of multiple parallel microchannels in a two-layer stacked microfluidic device. The fabrication of this device is simple without the need of a precise alignment of the two layers. The number of layers and the number of microchannels in each layer can thus be further increased for a potentially high throughput electrokinetic particle and cell manipulations.

## 1. Introduction

Electric field-driven flow is the transport method of choice in microfluidic devices over traditional pressure-driven flow due to its more favorite scaling with microchannel dimensions and easier operation with no mechanical connections [1,2,3,4,5]. It moves liquids by electroosmosis and charged samples (e.g., molecules, viruses, particles, cells, etc.) by electrophoresis [6,7]. The combination of these two motions gives the so-called electrokinetic motion, which possesses an essentially plug-like velocity profile in typical microchannels and thus can be precisely controlled [8,9,10]. However, electrokinetic manipulation of particles and cells suffers from an intrinsic drawback of low throughput due to its linear dependence of the fluid permittivity and particle/wall zeta potentials that usually have very small values [11,12]. While the increase of electric field can partially mitigate this problem, the throughput of electrokinetic manipulation is still limited because large electric fields can cause severe adverse effects such as Joule heating [13,14] and electrical damages [15,16] on both the sample and the microfluidic device itself.

We demonstrate in this work that the throughput of electrokinetic manipulation of particles and cells can be significantly increased by a parallel operation in multiple microchannels of a two-layer stacked microfluidic device. A similar idea of using multiple parallel channels has been recently developed to increase the signal-to-noise ratio in electric current monitoring for electroosmotic flow measurement under low ionic strengths [17]. It has also been demonstrated for achieving a high throughput in many other applications [18,19,20,21,22,23,24]. We utilize our recently developed reservoir-based dielectrophoresis (rDEP) technique [25,26] to implement a continuous concentration and separation of particles and cells inside the central reservoir of the stacked microfluidic device. A numerical model is also developed to simulate the electrokinetic particle and cell manipulations.

## 2. Experiment

### 2.1. Microfluidic Device Fabrication

**Figure 1:**
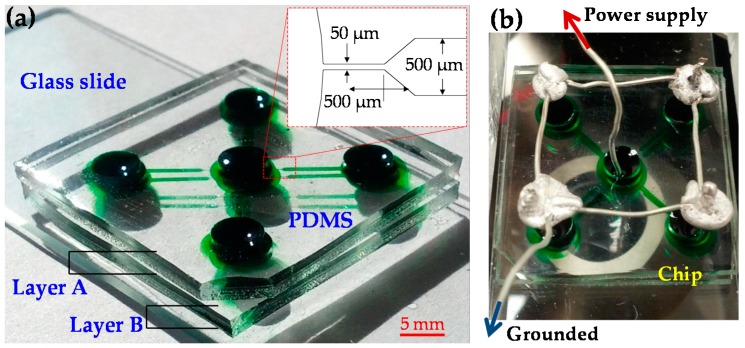
(**a**) Picture of the stacked PDMS/glass microfluidic device (reservoirs and microchannels are filled with green food dye for clarity) used in experiments, which consists of two PDMS layers with four 5-mm-long straight rectangular microchannels each; (**b**) Electrical connections for the stacked microfluidic device.

Figure 1a shows a picture of the microfluidic device fabricated for our experiments. This device consists of two stacked layers of polydimethylsiloxane (PDMS) slabs on top of a glass slide. Each PDMS layer has four straight rectangular microchannels that are arranged in the radial direction of the central inlet reservoir (for the supply of particles and cells), and each has an independent outlet reservoir. The microchannels in each layer of PDMS were fabricated using the standard soft lithography technique as described elsewhere [25]. They are each 500-μm-wide and 5-mm-long overall with a tapered 50-μm-wide, 500-μm-long constriction at the central reservoir-microchannel junction. This design is made to locally enhance the electric field and its gradients such that the electric voltage for particle and cell manipulations via rDEP can be reduced. All microchannels are of rectangular shape with a uniform 40-μm depth. The first layer of PDMS, i.e., Layer B in Figure 1a, was bonded to the glass slide after plasma treating (PDC-32G, Harrick Scientific, Ithaca, NY, USA), where no alignment was needed. The second layer of PDMS, i.e., Layer A in Figure 1a, was then plasma-bonded to the top of the first layer after the equal-sized reservoirs in each layer were aligned using only the naked eye. No precise alignment of the two layers is needed in this step because the electrokinetic manipulation of particles and cells at every central reservoir-microchannel junction takes place on its own and has no cross-talk with that at any other junctions.

### 2.2. Particle and Cell Solutions Preparation

Spherical polystyrene particles of 5 μm in diameter (Sigma-Aldrich, St. Louis, MO, USA) were used to demonstrate the electrokinetic trapping and concentration at the central reservoir-microchannel junctions via rDEP. They were re-suspended into 1 mM phosphate buffer to a final concentration of around 10^6^ particles per milliliter. The electric conductivity of the buffer solution was measured to be 210 μS/cm. In the electrokinetic separation experiment, 3-μm-diameter spherical polystyrene particles (Sigma-Aldrich) were mixed into the 5-μm particle solution at a similar final concentration. Tween 20 (Fisher Scientific, Waltham, MA, USA) was added to both particle suspensions at 0.1% *v*/*v* for suppressing the aggregation of particles and their adhesions to channel walls.

Yeast cells (*Saccharomyces cerevisiae* ATCC 9763) were procured from American Type Culture Center (Manassas, VA, USA), and cultivated in yeast-mold (YM) agar plates/yeast-mold broth at 30 °C. Briefly, a single colony of fresh yeast grown on a YM agar plate was used for inoculating YM broth (15 mL) in a sterile tube. The cells were grown at 30 °C under shaking conditions (250 rpm) for 24 h, and then centrifuged at 5000× *g* for 5 min before being re-suspended into 1× sterile phosphate buffer saline (PBS) for storage in a refrigerator. Prior to tests, yeast cells were washed/centrifuged with PBS at least 3 times and finally re-suspended in 1 mM phosphate buffer to a concentration of around 10^6^ cells per milliliter. They were mostly round with a measured diameter ranging from 4 to 8 μm. Tween 20 was also added to the cell suspension at 0.1% *v*/*v* to help dispersing cells, which did not cause visible lysis of the suspended yeast cells.

### 2.3. Particle/Cell Control and Visualization

The electrokinetic transport and manipulation of particles and cells in the stacked microfluidic device was attained by imposing DC-biased AC electric fields across all microchannels. The DC-biased AC voltages were imposed upon the platinum electrode (Fisher Scientific, Hampton, NH, USA) in the central inlet reservoir and supplied by a function generator (33220 A, Agilent Technologies, Santa Clara, CA, USA) in conjunction with a high-voltage amplifier (609E-6, Trek, Medina, NY, USA). The platinum electrodes in all four outlet reservoirs (see Figure 1) were grounded as viewed from Figure 1b. The experiment was run at 20 °C. The AC voltages had a sine waveform with a fixed frequency of 1 kHz. The motions of particles and cells were monitored through an inverted microscope (Nikon Eclipse TE2000U, Nikon Instruments, Lewisville, TX, USA) and recorded with a CCD camera (Nikon DS-Qi1Mc, Nikon Instruments Inc., Lewisville, TX, USA) at a frame rate of 15 per second. The focal point of the microscope objective lens was moved around horizontally and adjusted vertically to visualize the particle and cell behaviors at every central reservoir-microchannel junction in each PDMS layer. The obtained digital images were processed in the Nikon imaging software (NIS-Elements AR 3.22, Nikon Instruments Inc.). The particle and cell streak images were each obtained by superimposing a sequence of snapshot images.

## 3. Theory

### 3.1. Electrokinetic Particle and Cell Manipulation via rDEP

**Figure 2:**
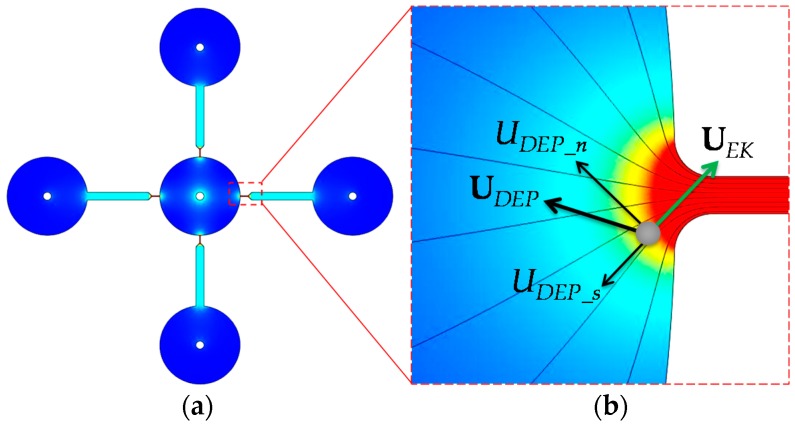
(**a**) Electric field contour in the four parallel microchannels of one PDMS layer of the stacked microfluidic device (see Figure 1). (**b**) Velocity analysis for a particle or cell at the central inlet reservoir-microchannel junction, where the electrokinetic motion, *U_EK_*, transports the particle or cell into the microchannel while the negative dielectrophoretic motion, *U_DEP_*, directs it towards the reservoir (by the streamwise component, *U_DEP_s_*) and the centerline of the microchannel (by the cross-stream component, *U_DEP_n_*). The thin lines represent the electric field lines or equivalently the fluid streamlines in the absence of the particle [30]. The darker background color indicates a larger electric field magnitude. The small circle at the center of the reservoir on the left plot indicates an iso-potential electrode surface.

The theory of rDEP has been detailed in our earlier study [27]. Briefly, due to the size difference between a reservoir and a microchannel, electric field gradients are inherently formed at each junction between the central inlet reservoir and the four microchannels in each PDMS layer as shown in Figure 2. A dielectrophoretic force is thus induced, which acts upon the incoming particles or cells and directs them back towards the central reservoir under DC and low-frequency (<100 kHz) AC electric field fields [16,28]. The resulting motion negative dielectrophoretic, *U_DEP_*, for spherical particles or cells of diameter *d* is written as [26,27]

(1) $$U_{DEP}=-\frac{\varepsilon d^{2}}{24\eta }\nabla E^{2},$$

where ε is the permittivity of the suspending medium, η is the dynamic viscosity of the suspending medium, and *E* is the electric field. Note that the Clausius–Mossotti (CM) factor has been assumed to be approximately −0.5 in Equation (1). This is because polystyrene particles and biological cells, which appear insulating in DC and low-frequency AC electric fields [16,28], have a much smaller electric conductivity than the suspending buffer solution in our experiments [29]. As illustrated by the particle velocity analysis in Figure 2, the streamwise component of the negative *U_DEP_*, i.e., *U_DEP_s_*, is against the electrokinetic motion of the particle, *U_EK_*. Since *U_EK_* is a linear function of the DC field component only, the increase in electric field, especially the AC component, will be able to make *U_DEP_s_* greater than *U_EK_*, yielding an electrokinetic trapping and concentration of particles and cells inside the reservoir [25,26,27]. Moreover, as *U_DEP_* is a second-order function of particle diameter while *U_EK_* has only a weak dependence, larger particles can be trapped more easily than smaller ones, enabling a selective concentration and separation of particles and cells at the reservoir-microchannel junction [27]. In addition, the cross-stream component of *U_DEP_*, i.e., *U_DEP_n_*, deflects particle and cells towards the channel centerline and produces a focusing effect [26,27]. We utilize *U_DEP_s_* to demonstrate in a stacked microfluidic device the electrokinetic concentration and separation of particles and cells at a significantly enhanced throughput in this work.

### 3.2. Numerical Simulation

To predict the electrokinetic manipulation of particles and cells in the parallel-operating microchannels of the stacked microfluidic device, we developed a 2D numerical model in COMSOL (Burlington, MA, USA) that covered all the reservoirs and microchannels in one layer of PDMS (see Figure 1). The Particle Tracing function was used to compute the trajectories of particles or cells that were released from different positions in the central inlet reservoir. The particle or cell velocity, *U_p_*, is the vector addition of *U_EK_* (due to the DC field component) and *U_DEP_* (due to both the DC and AC field components),

(2) $$U_{p}=\mu_{EK}E_{DC}+\lambda_{c}\mu_{DEP}\left(1+\alpha^{2}\right)\nabla E_{DC}^{2},$$

where μ*_EK_* is the electrokinetic particle mobility, ***E****_DC_* is the DC field component, λ*_c_* is the correction factor for particle size effects on DEP [31,32], μ*_DEP_* = −ε*d*^2^/24η is the dielectrophoretic particle mobility, and α is the AC to DC electric field ratio that is equal to the applied AC to DC voltage ratio. In the simulation, μ*_EK_* was determined by tracking the motions of individual particles or cells in the main-body of the microchannel under a small DC field where both the DEP and Joule heating effects are negligible [25,26,27]. We found μ*_EK_* = 3.3 × 10^−8^ m^2^/(V·s) for 5-μm and 3-μm polystyrene particles, and μ*_EK_* = 2.0 × 10^−8^ m^2^/(V∙s) for live yeast cells. It is important to note that the electroosmotic fluid flow pattern in the parallel microchannels is similar to the electric field distribution [30], whose influence on the particle and cell motions has been accounted for by μ*_EK_* in Equation (2) as a vector addition of electroosmotic mobility and electrophoretic mobility [4,10,12]. The influence from the wall frictional force has been considered by both μ*_EK_* and μ*_DEP_* in Equation (2). In addition, as the electroosmotic flow (and hence the electrokinetic particle motion) has a plug-like velocity profile, the cross-section of the microchannels has an insignificant influence on the particle and cell motions.

The DC electric field in Equation (2) was solved from Laplace equation with insulating and iso-potential boundary conditions on the walls and electrodes (see the small circle at the center of each reservoir in Figure 2), respectively [27]. The model equation was discretized using the finite element method and solved in an unstructured triangular mesh with a second-order accuracy. This numerical method has been validated in previous studies [10]. For the second term of *U_p_* on the right hand side of Equation (2), the dielectrophoretic mobility was calculated from the definition of μ*_DEP_* with ε = 6.9 × 10^−10^ C/(V·m) and η = 0.001 kg/(m·s). The correction factors were set to 0.6 and 0.8 for 5-μm and 3-μm particles, and 0.5 for yeast cells, which are consistent with the values used in our earlier studies [33,34] and found to match the experimental results well. Note that, although the yeast cells in our experiment have a size ranging from 4 to 8 μm, they were assumed spherical with an average diameter of 6 μm in the model. This treatment has been demonstrated to reasonably predict the experimental observation [10,25,33,34] because the electric field, which is identical in the experiment and simulation, is large enough to ensure that even the smallest cells can be sufficiently manipulated.

## 4. Results and Discussion

### 4.1. Electrokinetic Parallel Concentration of 5-μm Polystyrene Particles

**Figure 3:**
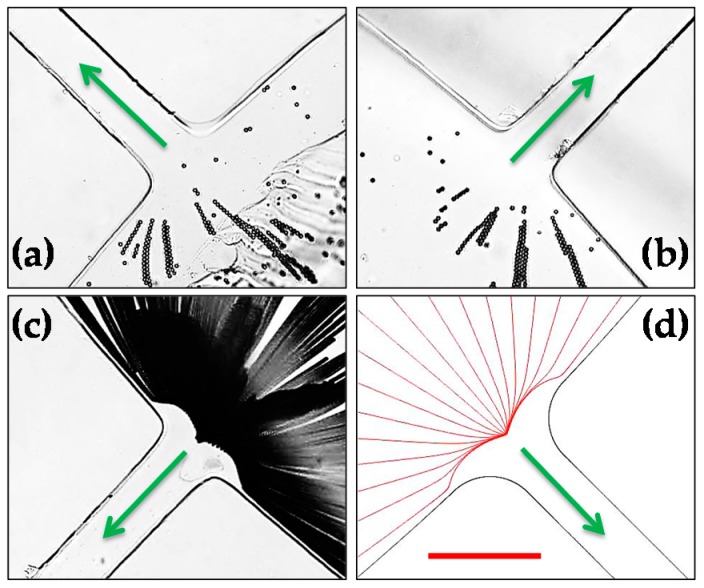
Electrokinetic trapping and concentration of 5-μm-diameter polystyrene particles inside the central inlet reservoir of the stacked microfluidic device under the application of a 50-V DC-biased 500-V AC voltage: (**a**) snapshot image in Layer A (see Figure 1); (**b**) snapshot image in Layer B; (**c**) superimposed image at a different reservoir-microchannel junction of Layer A; and (**d**) numerically predicted trajectories at any reservoir-microchannel junction. The arrows indicate the fluid and particles moving directions from the central inlet reservoir into the parallel microchannels. The scale bar represents 100 µm and applies to all images.

Figure 3 shows the electrokinetic trapping and concentration of 5-μm polystyrene particles inside the central inlet reservoir of the stacked microfluidic device under a 50-V DC-biased 500-V AC voltage. The two snapshot particle images in Figure 3a,b were taken from the PDMS layers A and B (see Figure 1), respectively, while the superimposed image in Figure 3c was from a different reservoir-microchannel junction in Layer A. Consistent with our earlier studies in a single-microchannel device [26,27], the trapped particles were observed to first form chains and then clusters before the entrance of every microchannel in the stacked microfluidic device while at a 700% higher throughput. With the further increase of the number of parallel-operating microchannels, this wholly in-reservoir operation can potentially be used for a high-throughput pre-concentration and filtration of various types of particles and cells. Figure 3d shows the numerically predicted particle trajectories, which agree with the superimposed particle image in Figure 3c well.

### 4.2. Electrokinetic Parallel Separation of 5-μm and 3-μm Polystyrene Particles

**Figure 4:**
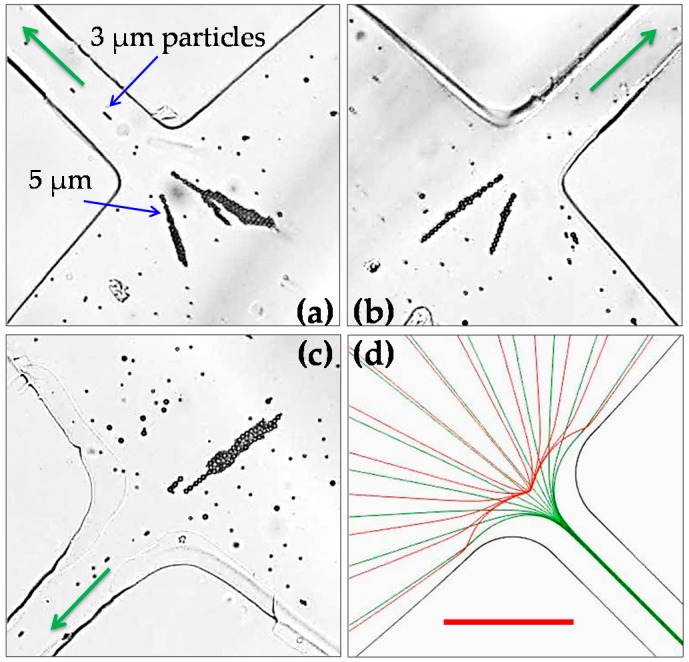
Electrokinetic concentration and separation of 5-μm-diameter polystyrene particles from 3-μm polystyrene particles at different central reservoir-microchannel junctions of the stacked microfluidic device under a 50-V DC-biased 500-V AC voltage: (**a**) snapshot image in Layer A (see Figure 1); (**b**) snapshot image in Layer B; (**c**) snapshot image at a different reservoir-microchannel junction of Layer A; and (**d**) numerically predicted trajectories at any reservoir-microchannel junction. The arrows indicate the fluid and particle moving directions during the separation. The scale bar represents 100 µm and applies to all images.

Figure 4 shows the electrokinetic separation of 5-μm and 3-μm polystyrene particles at the central reservoir-microchannel junctions of the stacked microfluidic device. Under the application of a 50-V DC-biased 500-V AC voltage, 5-μm particles become trapped and concentrated inside the central reservoir, which is consistent with the observation in Figure 3. In contrast, 3-μm particles can travel into the microchannels in the form of an apparently narrowed stream. This is because the streamwise dielectrophoretic motion, *U_DEP_s_*, of the smaller particles induced at the junctions is not strong enough to overcome the particle size-independent electrokinetic motion, *U_EK_*. Meanwhile, however, the cross-stream dielectrophoretic motion, *U_DEP_n_*, takes effect to deflect the smaller particles towards the centerline of every microchannel. With this observed electrokinetic focusing, concentration and separation behaviors of the particle mixture at every central reservoir-microchannel junction of the stacked microfluidic device are again consistent with those in a single-microchannel device in our earlier study [26,27]. Moreover, these experimental observations are all reasonably predicted by the numerical model.

### 4.3. Electrokinetic Parallel Concentration of Yeast Cells

**Figure 5:**
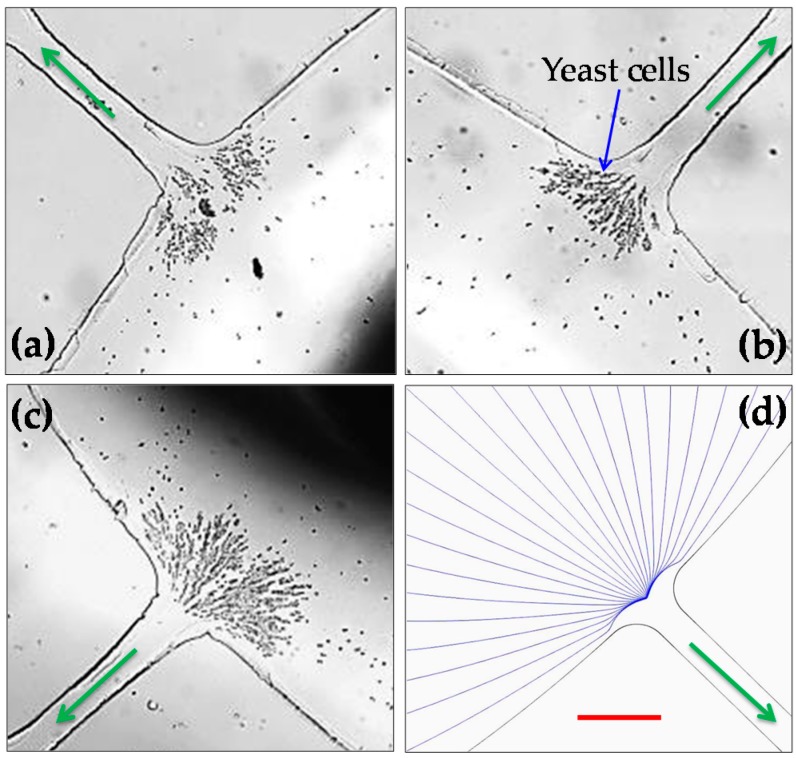
Electrokinetic concentration of live yeast cells at different central reservoir-microchannel junctions of the stacked microfluidic device under a 25-V DC-biased 250-V AC voltage: (**a**) snapshot image in Layer A (see Figure 1); (**b**) snapshot image in Layer B; (**c**) snapshot image at a different reservoir-microchannel junction of Layer A; and (**d**) numerically predicted trajectories at any reservoir-microchannel junction. The arrows indicate the fluid and cell moving directions. The scale bar represents 100 µm and applies to all images.

Figure 5 demonstrates the application of the stacked microfluidic device to electrokinetic concentration of yeast cells inside the central inlet reservoir. To reduce the potential electrical damages to cells as well as the negative Joule heating effects on the cells, we used a smaller DC bias voltage of 25 V to drive the cell suspension. We found that an AC voltage of 200 V is sufficient to achieve the trapping and concentration of yeast cells at the reservoir-microchannel junction by rDEP. No apparent increase in the electric current was monitored, indicating insignificant Joule heating effects during this experiment [13,14]. Moreover, the concentrated yeast cells were extracted from the central inlet reservoir using a digital pipette and tested with methylene blue. More than 90% of the cells were found still live after enduring the electrokinetic trapping process. In addition, it is interesting to note that the trapped yeast cells tend to first form smaller irregular clusters and then larger clusters, which is different from the observed trapping pattern for polystyrene particles in Figure 3. This may be due to the irregular shape of yeast cells that can significantly complicate the cell–cell interactions in the electric field [29]. Our numerical model traces only single particles or cells, and hence is unable to predict the trapping pattern for both polystyrene particles in Figure 3 and yeast cells in Figure 5.

## 5. Conclusions

We have fabricated a two-layer stacked microfluidic device with four parallel microchannels in each layer using the custom-modified soft lithography method. Our recently developed rDEP technique has been used to demonstrate a significantly enhanced throughput for electrokinetic manipulation of particles and cells in this device, as compared with that in a single-microchannel device. As the electrokinetic manipulations take place wholly within the central inlet reservoir of this stacked device, the extraction of the concentrated and separated particles and cells is simplified. The process of the device fabrication is straightforward without the need to precisely align the two layers. The number of layers and the number of microchannels in each layer of this device can thus be further increased for a potentially high throughput. However, one potential problem with densely packed microchannels for electrokinetic manipulations is the increase in volumetric Joule heating effects [13,14], which is a problem that becomes more severe if the electrical conductivity of the suspending medium must not be low in order to keep the nature of bio-samples [15,16].
